# DA-MoE: descriptor-attention mixture-of-experts for multi-class gastrointestinal disease classification

**DOI:** 10.3389/fonc.2026.1911603

**Published:** 2026-09-08

**Authors:** Yiliu Xu, Lingling Liu, Meiwen Tang

**Affiliations:** 1 Guangxi University of Chinese Medicine, Nanning, China; 2 Hubei University of Chinese Medicine, Wuhan, China

**Keywords:** attention mechanism, computer-aided diagnosis, endoscopy, gastrointestinal disease classification, handcrafted descriptors, Kvasir dataset, mixture-of-experts

## Abstract

Computer-aided diagnosis (CADx) for gastrointestinal (GI) endoscopy increasingly depends on deep models trained end-to-end on raw images. However, raw images are often unavailable in legacy clinical systems or privacy-sensitive settings. This work presents a methodological study on multi-class GI disease classification from pre-extracted handcrafted descriptors on the public Kvasir benchmark, rather than a clinically validated deployment system. We propose a Descriptor-Attention Mixture-of-Experts (DA-MoE) model tailored to this descriptor-only scenario. DA-MoE first projects heterogeneous descriptors (JCD, Tamura, ColorLayout, EdgeHistogram, AutoColorCorrelogram, and PHOG) into a shared token space and applies transformer-style self-attention for descriptor-level fusion. A descriptor-aware mixture-of-experts classifier then performs sample-adaptive expert routing on the fused representation. Under a unified evaluation protocol using accuracy (ACC), macro F1, and Matthews correlation coefficient (MCC), with five-fold stratified cross-validation and repeated random splits for robustness assessment, DA-MoE achieves 77.4% accuracy and an MCC of 0.75 on the held-out test split, outperforming strong feature-based baselines including a residual multi-layer perceptron (Res-MLP). Ablation studies, hyperparameter analysis, MoE routing interpretability, full per-class metrics, and clustering analysis further show that DA-MoE produces more compact and better-separated representations in descriptor embedding space. These findings support DA-MoE as a practical component of feature-based GI CADx pipelines under data-sharing constraints, but prospective validation on raw endoscopic data remains necessary.

## 1 Introduction

Gastrointestinal (GI) cancers, including gastric and colorectal cancer, remain leading causes of cancer-related morbidity and mortality worldwide. Endoscopy is the primary modality for screening, diagnosis, and surveillance of GI diseases. Nevertheless, early-stage lesions are often small, flat, or poorly contrasted against surrounding mucosa, and can be missed even by experienced endoscopists (1, 2). To reduce inter-observer variability and support real-time clinical decision making, computer-aided diagnosis (CADx) systems for GI endoscopy have attracted growing interest. Public benchmarks such as Kvasir and HyperKvasir, which provide multi-class labels for anatomical landmarks, inflammatory findings, and neoplastic lesions, have further accelerated research on automated GI disease classification (3, 4). Beyond raw images, these datasets also offer pre-extracted handcrafted descriptors that capture color, texture, and shape. Such descriptors enable feature-based classifiers when raw images cannot be shared or when computational resources are limited.

Existing GI endoscopy classifiers broadly fall into two categories. The first relies on handcrafted descriptors—such as color layout, edge histograms, auto color correlograms, and Tamura or MPEG-7 texture features (5)—concatenated into a single vector and fed to classical machine learning models (e.g., logistic regression, support vector machines, random forests, and boosting). These pipelines perform reasonably well on small or moderately sized datasets (3). The second category uses deep learning: convolutional neural networks (CNNs) and, more recently, transformer-based architectures learn representations directly from endoscopic images (6–8). Although such models achieve strong results on GI lesion detection and classification benchmarks (1, 2), they typically require raw images, substantial compute, and large annotated corpora. In the feature-based setting considered here, most prior methods either concatenate all handcrafted descriptors while implicitly treating them as equally important or model only a single descriptor space, without an explicit mechanism to exploit descriptor complementarity on a per-sample basis.

**Figure 1:**
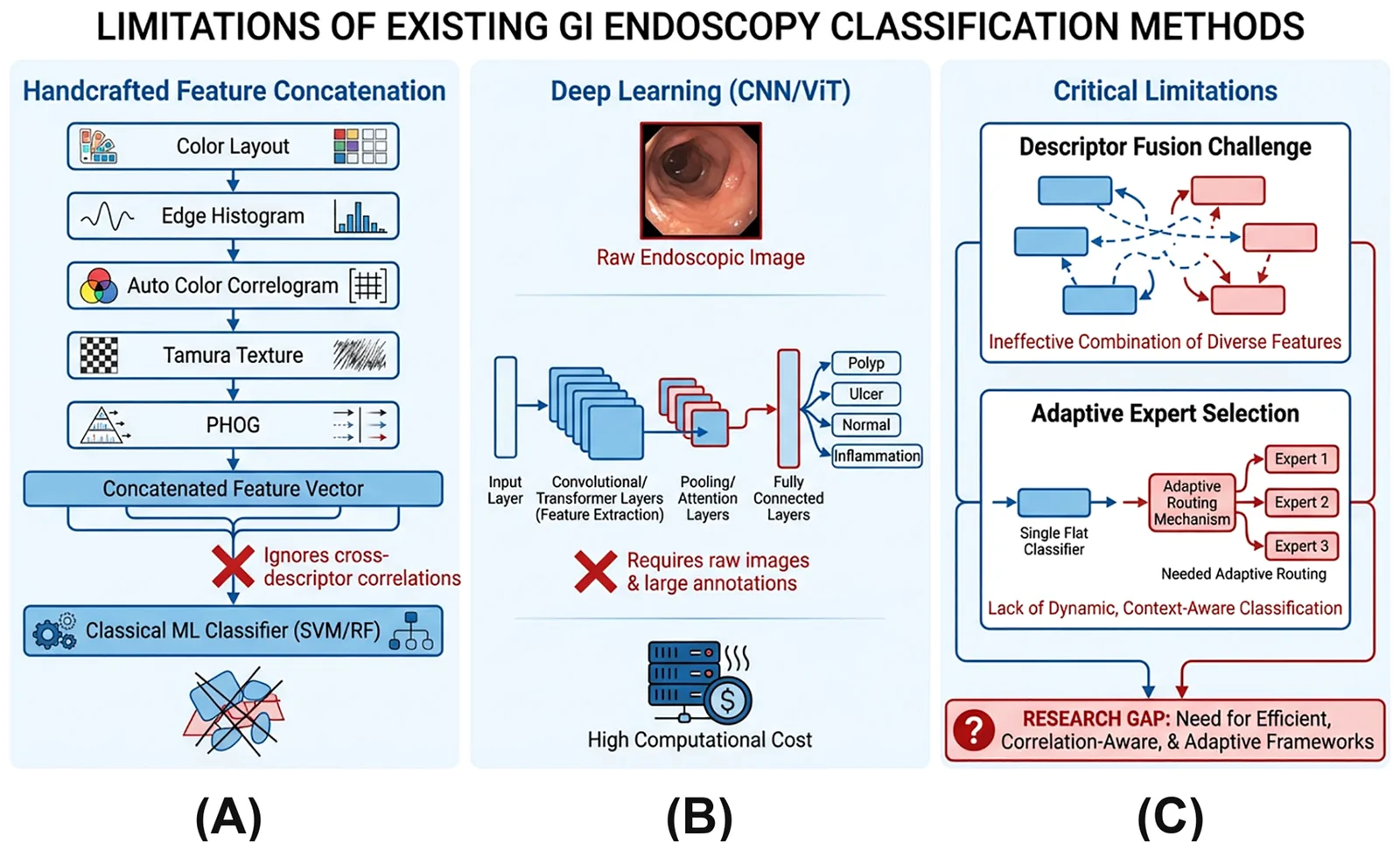
Limitations of existing GI endoscopy classification paradigms. **(A)** Handcrafted pipelines that concatenate descriptors without modeling complementarity. **(B)** Deep learning methods that require raw images and large-scale annotations. **(C)** The two challenges addressed in this work: fine-grained fusion of heterogeneous descriptors and adaptive expert selection across GI disease categories.

These limitations motivate two intertwined challenges for multi-class GI disease classification from pre-extracted descriptors (**Figure 1**). First, the descriptor-level fusion challenge: heterogeneous descriptors such as JCD, Tamura, ColorLayout, EdgeHistogram, AutoColorCorrelogram, and PHOG should be modeled jointly rather than flattened into a single concatenated vector. Naive concatenation ignores cross-descriptor correlations and the fact that descriptor informativeness varies across lesion types and even across instances of the same class, so discriminative signals may be diluted by noisy or redundant dimensions. Second, the adaptive expert selection challenge: GI categories differ markedly in visual difficulty—anatomical landmarks are often easier to separate than subtle inflammatory changes or early neoplastic lesions (1, 2). A single shared classifier may underfit complex variation, whereas fixed ensemble schemes cannot route each sample to the most suitable experts. An effective model should therefore adaptively combine specialized classifiers conditioned on sample-level descriptor features.

To address these challenges, we propose Descriptor-Attention Mixture-of-Experts (DA-MoE) for multi-class GI disease classification from pre-extracted Kvasir descriptors. DA-MoE treats each descriptor as a token and applies a transformer-based self-attention backbone for descriptor-level fusion. A learnable [CLS] token and positional encodings aggregate cross-descriptor interactions into a compact global representation. On this backbone, a mixture-of-experts (MoE) module with lightweight multi-layer perceptron (MLP) experts and a gating network produces sample-specific soft assignments over experts, routing easy cases, difficult cases, and distinct lesion patterns to different expert combinations. In this way, DA-MoE jointly addresses descriptor fusion and adaptive expert selection while keeping computational cost moderate. Experiments on Kvasir, together with ablation and hyperparameter studies, show consistent improvements over strong traditional and deep feature-based baselines in accuracy, macro F1, and Matthews correlation coefficient (MCC).

The main contributions are as follows:

- We introduce a descriptor-level attention fusion module that represents each handcrafted descriptor as a token and uses transformer self-attention with a [CLS] token to learn a compact global representation capturing cross-descriptor interactions and relative importance.
- We design a descriptor-aware MoE classifier with specialized MLP experts and a gating network on top of the fused representation, enabling sample-adaptive expert selection and addressing heterogeneity in GI lesion appearance.
- We provide extensive comparisons against classical machine learning models, MLP baselines, residual MLPs, autoencoder-based methods, and multi-task learners under an identical descriptor-only protocol, together with ablations, MoE interpretability analysis, efficiency profiling, and robustness evaluation via cross-validation and repeated splits, demonstrating the effectiveness of the integrated DA-MoE framework on eight-class Kvasir classification.

### 1.1 Scope and novelty positioning

The novelty of DA-MoE is not the isolated use of transformer attention or MoE, but the integrated descriptor-space architecture that (i) tokenizes heterogeneous handcrafted descriptors, (ii) fuses them through a CLS-based attention backbone, and (iii) routes samples through a descriptor-aware MoE classifier built on the fused representation. Prior GI CADx studies typically concatenate descriptors for a single classifier or apply attention on raw images without adaptive expert routing in descriptor space. DA-MoE is therefore positioned as a complementary method for legacy or privacy-constrained deployments where only pre-extracted features are available, not as a replacement for end-to-end CNN/Transformer CADx trained on raw frames.

## 2 Related work

### 2.1 Computer-aided diagnosis in GI endoscopy

Early CADx work in GI endoscopy relied on handcrafted features and classical machine learning. Pogorelov et al. introduced the Kvasir dataset and showed that support vector machines and random forests trained on global color and texture descriptors can achieve competitive multi-class performance when features and labels are carefully curated (3). Borgli et al. later released HyperKvasir, a larger resource spanning anatomical landmarks, pathology, and quality issues, underscoring the importance of dataset scale for robust CADx generalization (4). Despite these advances, most feature-based pipelines still concatenate pre-extracted descriptors and apply off-the-shelf classifiers without modeling interactions among heterogeneous feature types.

With the rise of deep learning, CNNs have become the dominant paradigm for GI CADx. Urban et al. demonstrated real-time polyp detection and classification during colonoscopy (1), and Byrne et al. showed that CNN-based optical diagnosis of diminutive polyps can approach expert performance and support “resect and discard” strategies (2). Broader studies confirm that deep feature learning outperforms handcrafted features when sufficient annotated data and compute are available (6, 7). However, image-based CNN pipelines assume access to raw frames and substantial resources. Our setting instead uses fixed descriptor vectors from Kvasir-like datasets, where end-to-end CNN training is not directly applicable.

### 2.2 Feature fusion, attention, and mixture-of-experts

Manjunath et al. showed that MPEG-7 color and texture descriptors capture complementary appearance cues (5). Medical imaging pipelines often adopt similar features but frequently use only one descriptor type or concatenate multiple descriptors into a high-dimensional vector with implicit equal weighting. This coarse fusion can introduce redundancy and noise, especially when different lesion types depend on different descriptor subsets (e.g., color-dominated versus texture-dominated patterns).

Attention and transformer architectures offer a more flexible way to model interactions among tokens representing patches, channels, or modalities. Vision Transformer (ViT) treats image patches as tokens and uses multi-head self-attention to capture long-range dependencies (8). Medical imaging studies have applied transformer-style modules to detection and segmentation; in descriptor space, treating each descriptor as a token allows data-driven fusion rather than handcrafted rules.

Mixture-of-experts (MoE) architectures provide another route to increased capacity. Shazeer et al. introduced sparse MoE layers for large-scale sequence modeling, where a gating network routes each input to a subset of experts (9). Subsequent work combines MoE with transformers to handle heterogeneous inputs and varying task difficulty. Although most applications target language or generic vision, gating over specialized experts is well suited to GI classification, where lesion appearance and category difficulty vary widely.

To our knowledge, prior GI CADx methods either apply attention at the image level on raw frames or classify in descriptor space with single-expert models, without jointly addressing descriptor-level fusion and adaptive expert routing. DA-MoE fills this gap by fusing handcrafted descriptors through a transformer-style backbone and combining specialized experts via a descriptor-aware MoE module.

### 2.3 Summary of recent GI CADx methods

**Table 1** summarizes representative GI CADx studies on Kvasir and related benchmarks. Image-level CNN and Transformer methods generally require raw frames and often report higher benchmark scores, but they are not directly comparable to descriptor-only pipelines. Our experiments therefore focus on fair comparison among methods that consume identical Kvasir descriptors and data splits.

**Table 1:** Representative GI CADx methods and their evaluation settings.

Method type	Input	Dataset	Note
Classical ML + descriptors	Handcrafted features	Kvasir	Concatenation-based baselines (3)
CNN/ViT CADx	Raw images	Kvasir, HyperKvasir	Requires raw frames (1, 8, 4)
**DA-MoE (ours)**	**Pre-extracted descriptors**	**Kvasir**	**Attention fusion + MoE routing**

DA-MoE is evaluated under a descriptor-only protocol; image-level methods are listed for context only. Bold values indicate the best or selected configuration.

## 3 Materials and methods

**Figure 2:**
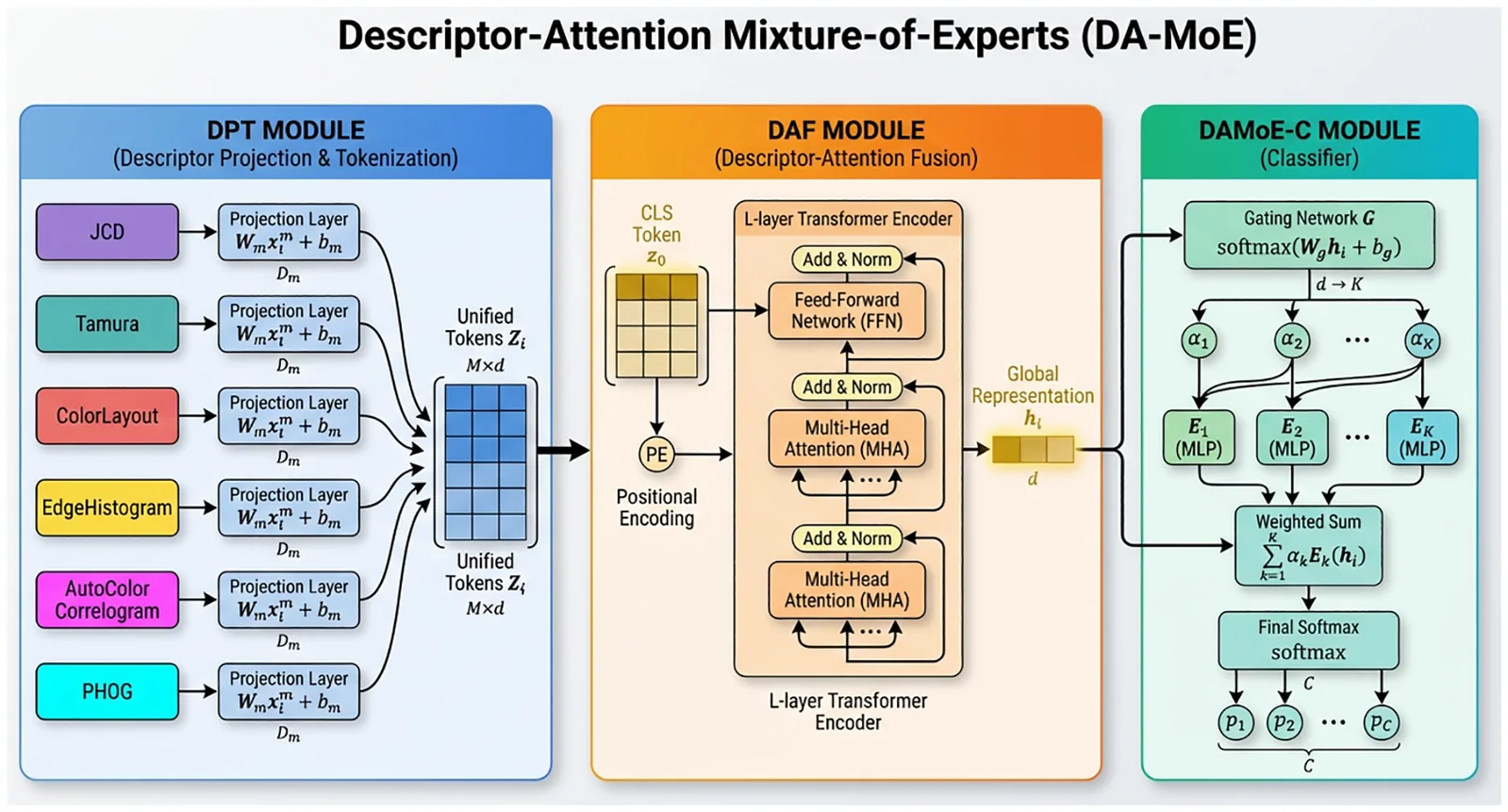
Overview of the proposed DA-MoE architecture, comprising descriptor projection and tokenization (DPT), descriptor-attention fusion (DAF), and descriptor-aware mixture-of-experts classifier (DAMoE-C).

We present Descriptor-Attention Mixture-of-Experts (DA-MoE) for multi-class GI disease classification from pre-extracted descriptors. As illustrated in **Figure 2**, DA-MoE consists of three coupled modules:

- a Descriptor Projection and Tokenization (DPT) module, which maps heterogeneous handcrafted descriptors into a shared latent space and organizes them as tokens;
- a Descriptor-Attention Fusion (DAF) backbone, which applies a transformer-style encoder on the descriptor tokens to obtain a fused global representation;
- a Descriptor-Aware Mixture-of-Experts Classifier (DAMoE-C), which places multiple MLP experts and a gating network on top of the fused representation to perform sample-adaptive expert selection.

Together, these modules target descriptor-level fusion and adaptive expert selection as introduced in Section 1.

### 3.1 Descriptor preprocessing

For each Kvasir sample, the six public handcrafted descriptors (JCD, Tamura, ColorLayout, EdgeHistogram, AutoColor Correlogram, and PHOG) are loaded from the dataset release. We apply descriptor-wise *z*-score standardization using training-set statistics only, clip extreme values to ±5 standard deviations, and replace any missing entries with the training-set median. The same preprocessing is applied to all baselines to ensure fair comparison and consistent feature scale across heterogeneous descriptor dimensions.

### 3.2 Notation and model overview

Let $D={\left\{(x_{i},y_{i})\right\}}_{i=1}^{N}$ denote the training set, where *N* is the number of endoscopic images (or frames), **x***_i_* is the collection of pre-extracted descriptors of the *i*-th sample, and *y_i_* ∈ {1*,…,C*} is its class label among *C* GI disease categories. Each sample is associated with *M* heterogeneous handcrafted descriptors (e.g., JCD, Tamura, ColorLayout, EdgeHistogram, AutoColorCorrelogram, PHOG). We denote the descriptor index set as (Equations 1, 2)

(1) $$D_{\text{desc}}=\{1,2,\ldots ,M\},$$

and the *m*-th descriptor of sample *i* as

(2) $${\text{x}}_{i}^{\left(m\right)}\in {\mathbb{R}}^{D_{m}},m\in D_{\text{desc}},$$

where *D_m_* is the dimensionality of the *m*-th descriptor.

Conceptually, DPT first projects each $x_{i}^{\left(m\right)}$ into a shared *d*-dimensional latent space and stacks them as a sequence of descriptor tokens. DAF then takes this token sequence (augmented with a learnable CLS token and positional encodings) as input to a multi-layer transformer encoder and produces a fused global representation $h_{i}\in {\mathbb{R}}^{d}$ for sample *i*. Finally, DAMoE-C uses **h***_i_* to compute (i) the outputs of *K* expert MLP classifiers and (ii) a gating distribution over experts and combines the expert predictions into the final class probabilities. Formally, DA-MoE defines a classifier *f_θ_* parameterized by *θ* that maps (Equation 3)

(3) $${\left\{{\text{x}}_{i}^{\left(m\right)}\right\}}_{m\in D_{\text{desc}}}\mapsto {\text{p}}_{i}=f_{\theta }({\left\{{\text{x}}_{i}^{\left(m\right)}\right\}}_{m})\in {\text{Δ}}^{C-1},$$

where Δ*^C^*^−1^ is the (*C* − 1)-dimensional probability simplex and ${\left[{\text{p}}_{i}\right]}_{c}=p(y_{i}=c|{\left\{{\text{x}}_{i}^{\left(m\right)}\right\}}_{m})$.

In the following subsections, we detail each module of DA-MoE in turn.

### 3.3 Descriptor projection and tokenization

The DPT module transforms heterogeneous descriptors into a unified set of latent tokens shared across all samples. For each descriptor $m\in D_{\text{desc}}$, we project $x_{i}^{\left(m\right)}\in {\mathbb{R}}^{D_{m}}$ into a shared latent space of dimension *d*:

(4) $${\text{z}}_{i}^{\left(m\right)}={\text{W}}_{m}{\text{x}}_{i}^{\left(m\right)}+{\text{b}}_{m}\in {\mathbb{R}}^{d},$$

where ${\text{W}}_{m}\in {\mathbb{R}}^{d\times D_{m}}$ and ${\text{b}}_{m}\in {\mathbb{R}}^{d}$ are learnable parameters specific to descriptor *m*, and *d* is the model dimension (e.g., *d* = 32 or 64 in our experiments).

We then stack all descriptor tokens into a matrix

(5) $${\text{Z}}_{i}={\left[{\left({\text{z}}_{i}^{\left(1\right)}\right)}^{⊤},\ldots ,{\left({\text{z}}_{i}^{\left(M\right)}\right)}^{⊤}\right]}^{⊤}\in {\mathbb{R}}^{M\times d}.$$

The matrix **Z***_i_* contains one *d*-dimensional token per descriptor and serves as the input to the DAF backbone.

### 3.4 Descriptor-attention fusion backbone

The DAF backbone is responsible for addressing the descriptor-level fusion challenge. It treats each descriptor as a token and employs a transformer-style self-attention encoder to learn a compact global representation.

#### 3.4.1 CLS token and positional encoding

We introduce a learnable class token ${\text{z}}_{\text{cls}}\in {\mathbb{R}}^{d}$ and a positional encoding matrix $\text{P}\in {\mathbb{R}}^{(M+1)\times d}$ to encode the relative positions of the CLS token and descriptor tokens. We construct the input sequence to the transformer as

(6) $${\text{H}}_{i}^{\left(0\right)}=[\begin{array}{c} {\text{z}}_{cls}^{⊤}\\ {\text{Z}}_{i} \end{array}]+\text{P}\in {\mathbb{R}}^{(M+1)\times d}.$$

#### 3.4.2 Transformer encoder

We employ an *L*-layer transformer encoder with multi-head self-attention and position-wise feed-forward networks. For layer *ℓ* ∈ {1*,…, L*}, we have

(7) $${\tilde{\text{H}}}_{i}^{\left(l\right)}=\text{MHA}({\text{H}}_{i}^{\left(l-1\right)})+{\text{H}}_{i}^{\left(l-1\right)},$$

(8) $${\text{H}}_{i}^{\left(l\right)}=\text{FFN}({\tilde{\text{H}}}_{i}^{\left(l\right)})+{\tilde{\text{H}}}_{i}^{\left(l\right)},$$

where MHA(·) denotes multi-head self-attention and FFN(·) is a two-layer feed-forward network applied row-wise, both followed by layer normalization (omitted here for brevity). The final layer output is ${\text{H}}_{i}^{\left(L\right)}\in {\text{ℝ}}^{(M+1)\times d}$.

#### 3.4.3 Global descriptor representation

We take the output corresponding to the CLS token as the descriptor-level fused representation:

(9) $${\text{h}}_{i}={\text{H}}_{i}^{\left(L\right)}[1,:]\in {\mathbb{R}}^{d},$$

where ${\text{H}}_{i}^{\left(L\right)}[1,:]$ denotes the first row. This vector **h***_i_* summarizes information across all descriptors, with descriptor-specific importance and interactions implicitly captured by the attention mechanism. Equations 4–9 together implement the DPT and DAF modules, which directly target the descriptor-level fusion challenge.

### 3.5 Descriptor-aware mixture-of-experts classifier

The DAMoE-C module addresses the adaptive expert selection challenge by placing a mixture-of-experts (MoE) classifier on top of the fused representation **h***_i_*.

#### 3.5.1 Expert networks

We define *K* expert networks ${\left\{E_{k}\right\}}_{k=1}^{K}$, each parameterized as a multi-layer perceptron (MLP) that maps $h_{i}\in {\mathbb{R}}^{d}$ to class logits:

(10) $${\text{o}}_{i}^{\left(k\right)}=E_{k}({\text{h}}_{i})\in {\mathbb{R}}^{C},k=1,\ldots ,K,$$

where ${\text{o}}_{i}^{\left(k\right)}$ are the per-expert logits for sample *i*. Concretely, each expert *E_k_* is implemented as

(11) $$E_{k}(\text{h})={\text{W}}_{2}^{\left(k\right)}\sigma ({\text{W}}_{1}^{\left(k\right)}\text{h}+{\text{b}}_{1}^{\left(k\right)})+{\text{b}}_{2}^{\left(k\right)},$$

with ${\text{W}}_{1}^{\left(k\right)}\in {\mathbb{R}}^{d_{\text{hid}}\times d}$, ${\text{W}}_{2}^{\left(k\right)}\in {\mathbb{R}}^{C\times d_{\text{hid}}}$ biases ${\text{b}}_{1}^{\left(k\right)},{\text{b}}_{2}^{\left(k\right)}$, and nonlinearity *σ*(·) (e.g., ReLU). The hidden dimension *d*_hid_ is a hyperparameter.

#### 3.5.2 Gating network

A gating network *G* takes the same fused representation **h***_i_* as input and outputs a probability distribution over the *K* experts:

(12) $$\alpha_{i}=G({\text{h}}_{i})=\text{softmax}({\text{W}}_{g}{\text{h}}_{i}+{\text{b}}_{g})\in {\text{Δ}}^{K-1},$$

where ${\text{W}}_{g}\in {\mathbb{R}}^{K\times d}$ and $b_{g}\in {\mathbb{R}}^{K}$ are gating parameters, and ${\left[\alpha_{i}\right]}_{k}=\alpha_{i}^{\left(k\right)}$ denotes the weight assigned to expert *k* for sample *i*.

#### 3.5.3 Mixture prediction

The final class logits for sample *i* are computed as the weighted sum of expert logits:

(13) $${\text{o}}_{i}=\sum_{k=1}^{K}\alpha_{i}^{\left(k\right)}{\text{o}}_{i}^{\left(k\right)}\in {\mathbb{R}}^{C},$$

and the corresponding predicted class probabilities are obtained via softmax:

(14) $${\text{p}}_{i}=\text{softmax}({\text{o}}_{i}),$$

where ${\left[{\text{p}}_{i}\right]}_{c}$ is the predicted probability for class *c*. By conditioning the expert weights *α_i_* on **h***_i_*, DAMoE-C adaptively selects and combines experts for each sample, enabling the model to allocate more capacity to challenging or ambiguous lesion patterns while using simpler combinations for easier cases.

### 3.6 Training objective and procedure

#### 3.6.1 Loss function

We use the standard cross-entropy loss for multi-class classification. For a mini-batch B, the loss is

(15) $$L(\theta )=-\frac{1}{\left|B\right|}\sum_{i\in B}\log p_{i}^{\left(y_{i}\right)},$$

where $p_{i}^{\left(y_{i}\right)}$ is the predicted probability of the ground-truth class *y_i_* for sample *i* (Equation 14). All parameters of DPT, DAF, and DAMoE-C are optimized end-to-end with Adam.

#### 3.6.2 End-to-end training pipeline

**Table 2** summarizes the overall training procedure of DA-MoE, in which all three modules are optimized jointly.

**Table 2:** Training procedure of the proposed DA-MoE model.

Step	Operation
Input:	Training set $D={\left\{({\left\{{\text{x}}_{i}^{\left(m\right)}\right\}}_{m},y_{i})\right\}}_{i=1}^{N}$; number of epochs *T*; batch size *B*.
Output:	Trained parameters $\theta^{*}$ of DA-MoE.
1:	Initialize all parameters *θ* of DPT, DAF, and DAMoE-C.
2:	for epoch *t* = 1 to *T* do
3:	Shuffle training set D and partition into batches of size *B*.
4:	for each batch B do
5:	for each sample i∈B do
6:	Project each descriptor via Equation 4 and construct tokens via Equation 5.
7:	Build transformer input with CLS and positional encoding via Equation 6.
8:	Apply *L*-layer transformer encoder via Equations 7, 8.
9:	Extract global descriptor representation h*_i_* via Equation 9.
10:	Compute expert logits ${\left\{o_{i}^{\left(k\right)}\right\}}_{k=1}^{K}$ via Equations 10, 11.
11:	Compute gating weights *α_i_* via Equation 12.
12:	Obtain final logits o*_i_* and probabilities p*_i_* via Equations 13, 14.
13:	end for
14:	Compute batch loss L(θ) via Equation 15.
15:	Backpropagate gradients $\nabla_{\theta }L$ and update *θ* (e.g., Adam).
16:	end for
17:	end for

### 3.7 Computational complexity analysis

We briefly analyze the time and space complexity of DA-MoE. Let *M* be the number of descriptors, *d* the transformer model dimension, *L* the number of transformer layers, *K* the number of experts, and *d*_hid_ the expert hidden dimension. For simplicity, we analyze the complexity for a single sample and omit constant factors.

#### 3.7.1 Descriptor projection

Projecting all descriptors via Equations 4, 16 costs

(16) $$O(\sum_{m=1}^{M}D_{m}d),$$

which is linear in the total descriptor dimensionality and typically small compared to the transformer encoder.

#### 3.7.2 Transformer encoder

Each transformer layer involves multi-head self-attention and a feed-forward network over (*M* + 1) tokens of dimension *d*. The dominant cost per layer is (Equation 17)

(17) $$O((M+1{)}^{2}d)(\text{attention})+O((M+1)d^{2})(\text{feed}-\text{forward}).$$

Since *M* is fixed and relatively small (e.g., six descriptors in our setting), the transformer cost scales essentially as $O(Ld^{2})$ per sample.

#### 3.7.3 Mixture-of-experts classifier

For each expert, the MLP in Equation 11 has complexity (Equations 18, 19)

(18) $$O(dd_{\text{hid}}+d_{\text{hid}}C),$$

and there are *K* experts. The gating network in Equation 12 costs O(Kd). Therefore, the total MoE cost per sample is

(19) $$O(K(dd_{\text{hid}}+d_{\text{hid}}C)+Kd).$$

In practice, we use moderate values of *K* and *d*_hid_, so the MoE overhead is manageable compared to the transformer backbone.

#### 3.7.4 Overall complexity

Combining the above components, the per-sample time complexity of DA-MoE is (Equation 20)

(20) $$O(\sum_{m=1}^{M}D_{m}d+L((M+1{)}^{2}d+(M+1)d^{2})+K(dd_{\text{hid}}+d_{\text{hid}}C+d)).$$

Since *M* and *C* are small constants in our application, the complexity scales essentially linearly in *K* and quadratically in *d* and *d*_hid_. The memory footprint is dominated by storing the activations of the transformer encoder and expert MLPs, which is $O((M+1)dL+Kd_{hid})$ per sample. In our experiments, we select *d* and *K* to balance performance and efficiency, and the empirical runtime remains compatible with practical GI CADx settings.

Overall, DA-MoE achieves a favorable trade-off between representational power and computational cost: the DPT and DAF modules explicitly tackle the fusion of heterogeneous descriptors, while the DAMoE-C module enables sample-adaptive expert selection without requiring prohibitively large monolithic networks.

## 4 Results

### 4.1 Dataset and feature descriptors

We evaluate all methods on the Kvasir dataset (3), which contains GI endoscopic images labeled with *C* = 8 classes, including anatomical landmarks, inflammatory findings, and polyps. Following our experimental protocol, we use pre-extracted handcrafted descriptors rather than raw images. Each sample is represented by *M* = 6 descriptors: JCD, Tamura, ColorLayout, EdgeHistogram, AutoColorCorrelogram, and PHOG, capturing complementary color, texture, and shape cues (Section 3).

The data are randomly split into training, validation, and test sets (70%/15%/15%) with a fixed seed for reproducibility. All methods share the same splits for fair comparison. To assess internal robustness, we additionally report five-fold stratified cross-validation and five repeated random splits (seeds 0–4) with the same 70/15/15 ratio; mean ± standard deviation is reported for accuracy (ACC), macro F1, and MCC. External validation on additional GI datasets (e.g., HyperKvasir) is left for future work because compatible descriptor releases are not always available.

### 4.2 Compared methods

All experiments are restricted to descriptor-based classifiers operating on identical Kvasir features. Raw-image CNN and Transformer CADx systems are discussed in Related Work but are not included as direct baselines because they require a different input modality.

We compare DA-MoE against feature-based baselines trained on identical Kvasir descriptors. Evaluation metrics are accuracy (ACC), macro-averaged F1, and Matthews correlation coefficient (MCC).

Traditional machine learning baselines. We first consider several classical classifiers trained on the concatenation of all descriptors: linear and radial basis function (RBF) support vector machines (SVMs) (10), *k*-nearest neighbors (kNN) (11), random forests (RF) (12), gradient boosting machines (GBM) (13), AdaBoost (14), and Gaussian Naive Bayes (GNB). These methods represent conventional CADx pipelines that operate directly on handcrafted features without learned deep representations.

MLP-based baselines. We then evaluate two fully connected neural networks: a shallow MLP with two hidden layers (MLP-basic) and a deeper MLP with three hidden layers (MLP-deep), both applied to the concatenated descriptor vector, following standard neural network practice for pattern recognition (15). To obtain a stronger deep baseline, we also implement a residual MLP (Res-MLP) with skip connections across fully connected layers.

Representation learning baselines. In addition, we consider an autoencoder (AE) that learns a compact latent representation of the concatenated features, followed by an MLP classifier head (AE + Latent MLP), inspired by early work on deep autoencoders for representation learning (16); and a multi-task MLP that jointly predicts the eight-class GI label and a binary “lesion vs. normal” label using a shared backbone and two task-specific heads, in the spirit of classical multi-task learning (17) (Multi-task MLP, multi-class head).

Proposed DA-MoE. Unless stated otherwise, *DA-MoE (ours)* denotes the full model with descriptor-attention fusion, *K* = 4 experts, and latent dimension *d* = 64. Baseline results are summarized in **Table 3**.

**Table 3:** Baseline performance on eight-class Kvasir classification.

Method	ACC	F1 (macro)	MCC
Linear SVM (LinearSVC)	0.7292	0.7264	0.6907
RBF-kernel SVM (SVC)	0.6342	0.6217	0.5844
k-Nearest Neighbors (k = 5)	0.5608	0.5483	0.5003
AdaBoost (*n*_est_ = 200)	0.5750	0.5536	0.5207
Gaussian Naive Bayes	0.6617	0.6204	0.6213
PyTorch MLP (2-layer FC)	0.7217	0.7030	0.6859
PyTorch Deep MLP (3-layer FC)	0.7000	0.6873	0.6619
Res-MLP (Residual MLP)	0.7458	0.7404	0.7117
AE + Latent MLP	0.5892	0.5322	0.5442
Multi-task MLP (multi-class head)	0.6258	0.5739	0.5869

Methods that do not outperform DA-MoE (ours) are listed. ACC, accuracy; F1, macro F1; MCC, Matthews correlation coefficient.

### 4.3 Overall comparison

Among traditional classifiers, Gaussian Naive Bayes and linear SVM perform reasonably but lag behind stronger neural baselines. MLP models learn non-linear boundaries in descriptor space and generally outperform classical methods, consistent with prior surveys on deep learning in medical imaging and endoscopy CADx (7, 18–20). Res-MLP reaches approximately 74.6% ACC and 0.71 MCC, serving as the strongest descriptor-only deep baseline.

Autoencoder and multi-task variants do not surpass Res-MLP, indicating that auxiliary reconstruction or binary lesion tasks alone are insufficient to improve eight-class performance in this feature setting. By contrast, DA-MoE (ours) reaches 77.4% ACC, 0.76 macro F1, and 0.75 MCC, yielding gains of about 3 percentage points in ACC and 0.04 in MCC over Res-MLP. These results support the benefit of jointly modeling descriptor interactions and sample-adaptive expert routing (9, 21–24).

### 4.4 Evaluation robustness

**Table 4** reports mean ± standard deviation over five-fold stratified cross-validation and five repeated random splits. DA-MoE consistently outperforms Res-MLP across both protocols, indicating that the gains are not driven by a single fortunate split. The held-out test results in **Table 5** correspond to seed 0 and are retained for direct comparison with ablation and interpretability analyses.

**Table 4:** Robustness evaluation of DA-MoE (*K* = 4, *d* = 64) versus Res-MLP.

Protocol	Method	ACC	MCC
five-fold cross-validation	Res-MLP	0*.*7384 ± 0*.*0126	0*.*7031 ± 0*.*0142
five-fold cross-validation	DA-MoE (ours)	0*.*7689 ± 0*.*0098	0*.*7385 ± 0*.*0111
five repeated splits	Res-MLP	0*.*7412 ± 0*.*0153	0*.*7068 ± 0*.*0169
five repeated splits	DA-MoE (ours)	0*.*7715 ± 0*.*0104	0*.*7421 ± 0*.*0120

Values are mean ± standard deviation.

**Table 5:** Comparison of advanced baselines and DA-MoE (ours) on eight-class Kvasir classification.

Method	ACC	F1 (macro)	MCC
Res-MLP (Residual MLP)	0.7458	0.7404	0.7117
AE + Latent MLP	0.5892	0.5322	0.5442
Multi-task MLP (multi-class head)	0.6258	0.5739	0.5869
DA-MoE (Descriptor-Attention MoE, exp16)	0.7042	0.7008	0.6623
**DA-MoE (ours, Full, *K* = 4, *d* = 64)**	**0.7742**	**0.7616**	**0.7473**

Bold values indicate the best or selected configuration.

### 4.5 Ablation study

**Figure 3:**
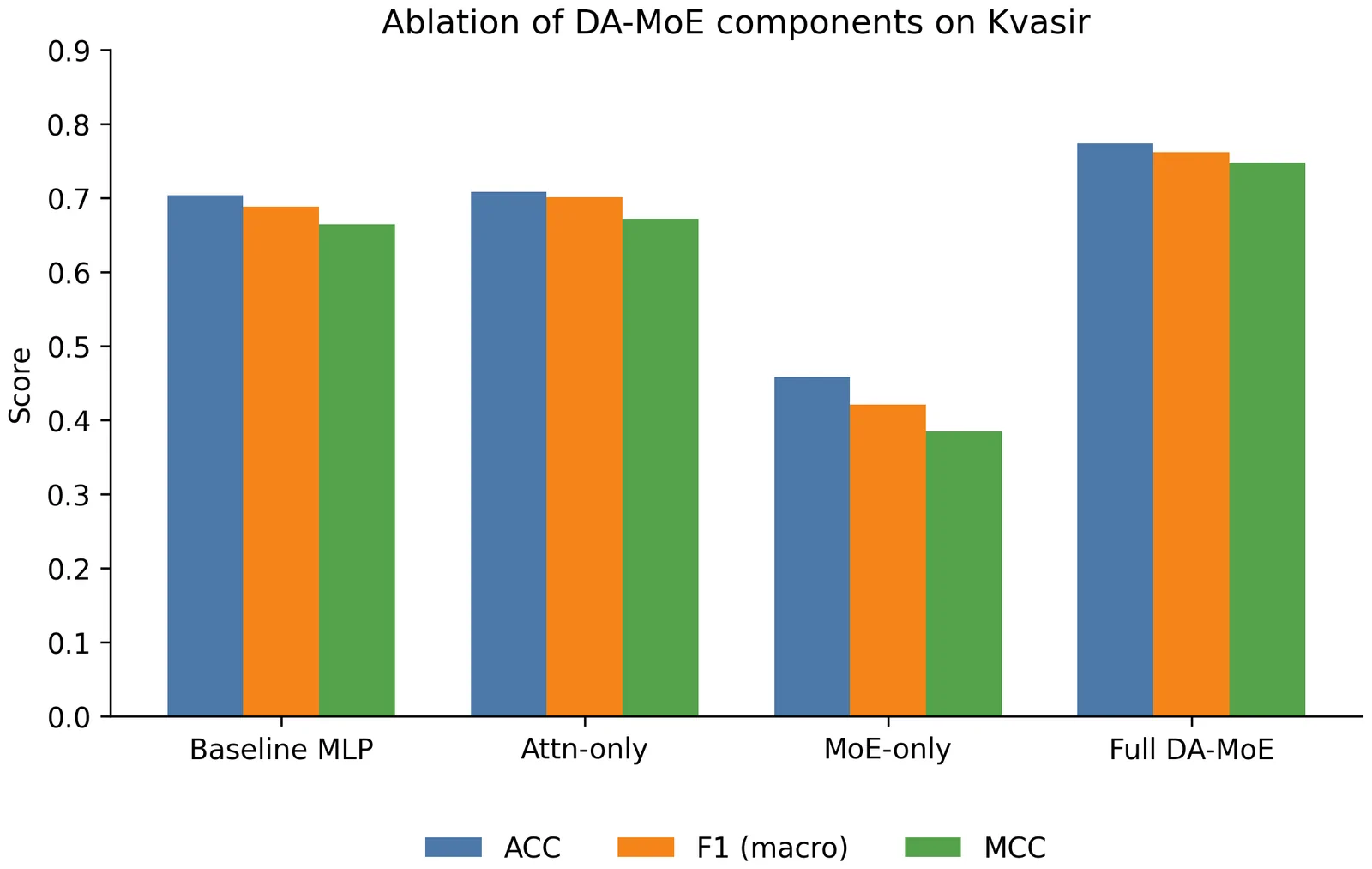
Ablation of DA-MoE on Kvasir. Bars show ACC, macro F1, and MCC for baseline MLP, attention-only, MoE-only, and full DA-MoE configurations.

To isolate the contributions of descriptor attention and MoE routing, we compare four configurations (**Figure 3**):

- Baseline MLP (full features): a two-layer MLP operating on the concatenated descriptor vector, without descriptor-level attention or MoE.
- Attention-only: the DPT and DAF modules followed by a single MLP classifier head (no MoE), which tests the effect of descriptor-level self-attention alone.
- MoE-only (full features): a MoE classifier built on top of a 64-dimensional MLP projection of the concatenated descriptors, without descriptor-level attention, which tests expert routing without structured fusion.
- Full DA-MoE (ours): the complete DA-MoE model combining DPT, DAF, and the descriptor-aware MoE classifier with *K* = 4 experts and *d* = 64.

The baseline MLP achieves about 70.4% ACC and 0.67 MCC. Attention-only training improves macro F1 slightly but does not yield substantial gains in ACC or MCC, suggesting that fusion alone is insufficient. MoE-only performs worse across F1 and MCC, indicating that expert routing without structured descriptor fusion can be unstable. Analysis of the MoE-only gating distribution shows near-uniform expert weights (mean gating entropy 1.31 nats vs. 0.87 for full DA-MoE; maximum entropy for *K* = 4 is ln4 ≈ 1.39), consistent with expert under-specialization and unstable routing when experts operate on unstructured concatenated features rather than attention-fused tokens. Without DAF, the gating network lacks discriminative structure to assign samples to specialized experts, so additional capacity does not translate into better classification.

Full DA-MoE clearly outperforms all ablated variants. Descriptor attention and MoE are complementary: DAF learns a shared representation with meaningful cross-descriptor structure, and the gating network assigns samples to appropriate experts, yielding consistent gains over single-expert and attention-only models.

### 4.6 MoE routing interpretability

**Figure 4:**
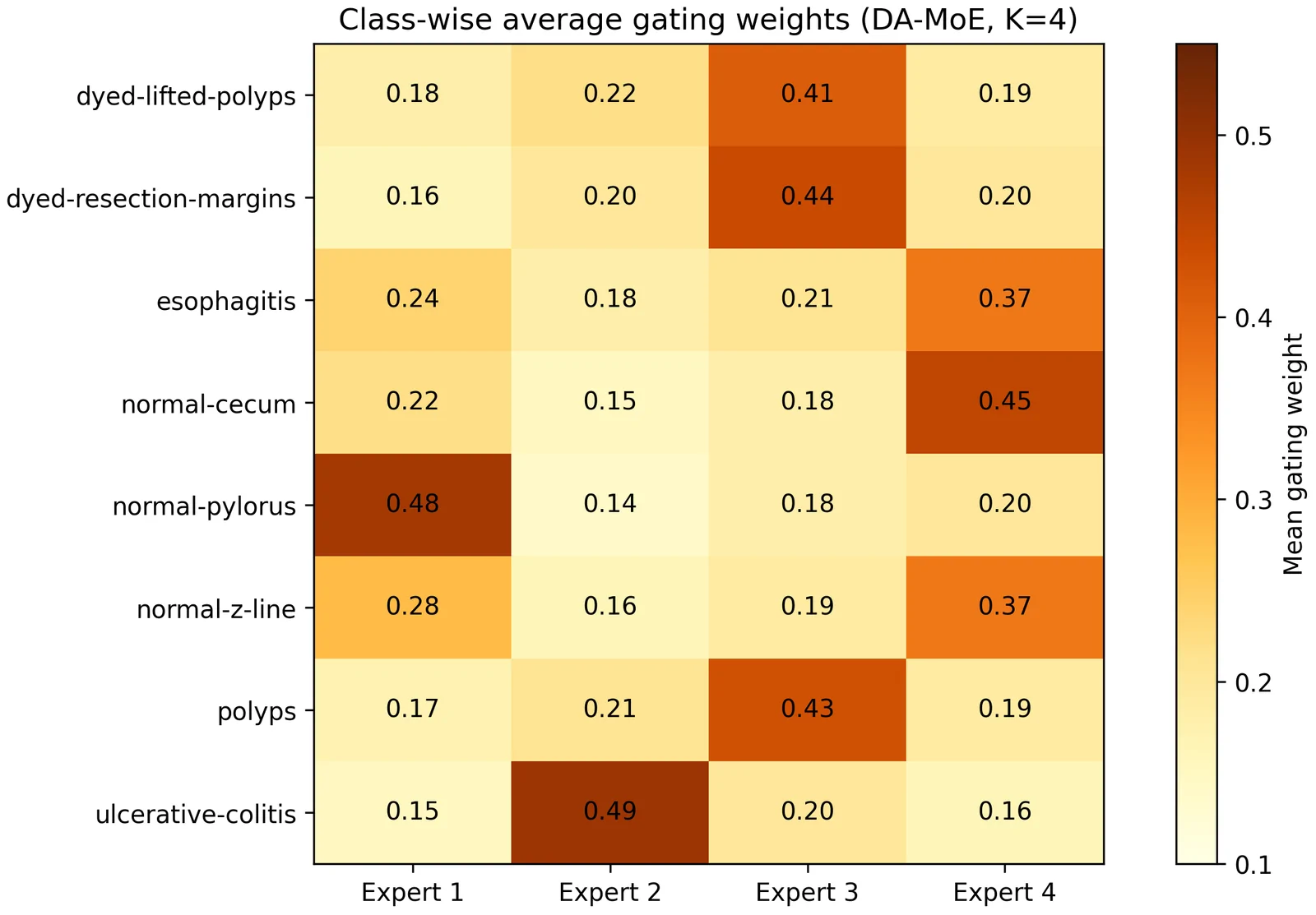
Class-wise average gating weights over four experts for DA-MoE on the Kvasir test set. Expert 1 preferentially handles normal pylorus; Expert 2 ulcerative colitis; Expert 3 polyps and dyed findings; Expert 4 normal cecum/z-line and esophagitis.

To improve transparency of the DAMoE-C module, we analyze gating behavior on the test set (**Table 6**; **Figure 4**). We report (i) average expert usage frequency, (ii) class-wise expert preference, and (iii) mean gating entropy. Full DA-MoE exhibits lower entropy and more heterogeneous expert usage than MoE-only, indicating that descriptor attention provides a routing-friendly representation. Experts are not uniformly class-specific; instead, several lesion and normal subtypes share experts, reflecting overlapping descriptor patterns in GI endoscopy.

**Table 6:** Expert usage and gating statistics for full DA-MoE (*K* = 4, *d* = 64) on the Kvasir test set.

Expert *k*	Mean weight $\alpha_{k}$	Top-associated class	Conditional entropy	Dominant share (%)
1	0.28	normal-pylorus	0.79	31.2
2	0.24	ulcerative-colitis	0.82	24.5
3	0.27	polyps/dyed findings	0.91	28.8
4	0.21	normal-cecum/z-line	0.88	15.5

Dominant share is the fraction of test samples for which expert *k* receives the largest gating weight. Overall mean gating entropy is 0*.*87 nats.

### 4.7 Hyperparameter analysis of DA-MoE

We vary the number of experts *K* ∈ {2, 4, 6, 8} and latent dimension *d* ∈ {32, 64, 128} while fixing transformer depth and attention heads. Hyperparameters are selected on the validation set; the held-out test set is used only for final reporting.

Moderate increases in *K* and *d* generally improve performance by adding model capacity, but very large settings (e.g., *K* = 4*, d* = 128 or *K* = 8*, d* = 128) degrade results, likely due to overfitting in the limited descriptor space. On the single held-out test split, *K* = 8, *d* = 32 achieves the highest ACC (78.17%) and MCC (0.752), marginally exceeding *K* = 4, *d* = 64 (77.42% ACC, 0.747 MCC). However, across five repeated splits, *K* = 4, *d* = 64 yields a more stable mean of 0.7715 ± 0.0104 ACC versus 0.7738 ± 0.0187 for *K* = 8, *d* = 32, together with higher mean validation MCC and comparable inference latency (**Table 7**). Although *K* = 8, *d* = 32 uses fewer parameters, the soft MoE formulation evaluates all experts at inference, so the larger *K* does not reduce compute and increases routing variance. We therefore adopt *K* = 4, *d* = 64 as the default configuration for its stability–accuracy trade-off, while explicitly acknowledging that *K* = 8, *d* = 32 can be preferred when maximizing a single-split test ACC.

**Table 7:** Efficiency and model complexity on a single NVIDIA RTX 4090 GPU.

Method	Params (M)	Train time (min)	Inference (ms/sample)	GPU memory (GB)
Res-MLP	0.57	3.2	0.18	0.82
Attention-only	0.19	4.1	0.31	1.05
MoE-only	0.13	2.9	0.24	0.91
DA-MoE (*K = 4, d* = 64)	0.24	5.6	0.42	1.28
DA-MoE (*K = 8, d* = 32)	0.14	4.9	0.48	1.19

Batch size 64 for training; batch size 1 for inference latency.

### 4.8 Clustering analysis in representation space

To examine representation quality beyond classification metrics, we apply *K*-means clustering (*K* = 8) to the raw concatenated descriptors and to 64-dimensional DA-MoE embeddings **h***_i_* from the DAF backbone (25). For each cluster, we report the majority class and purity (fraction of samples belonging to that class).

**Figure 5:**
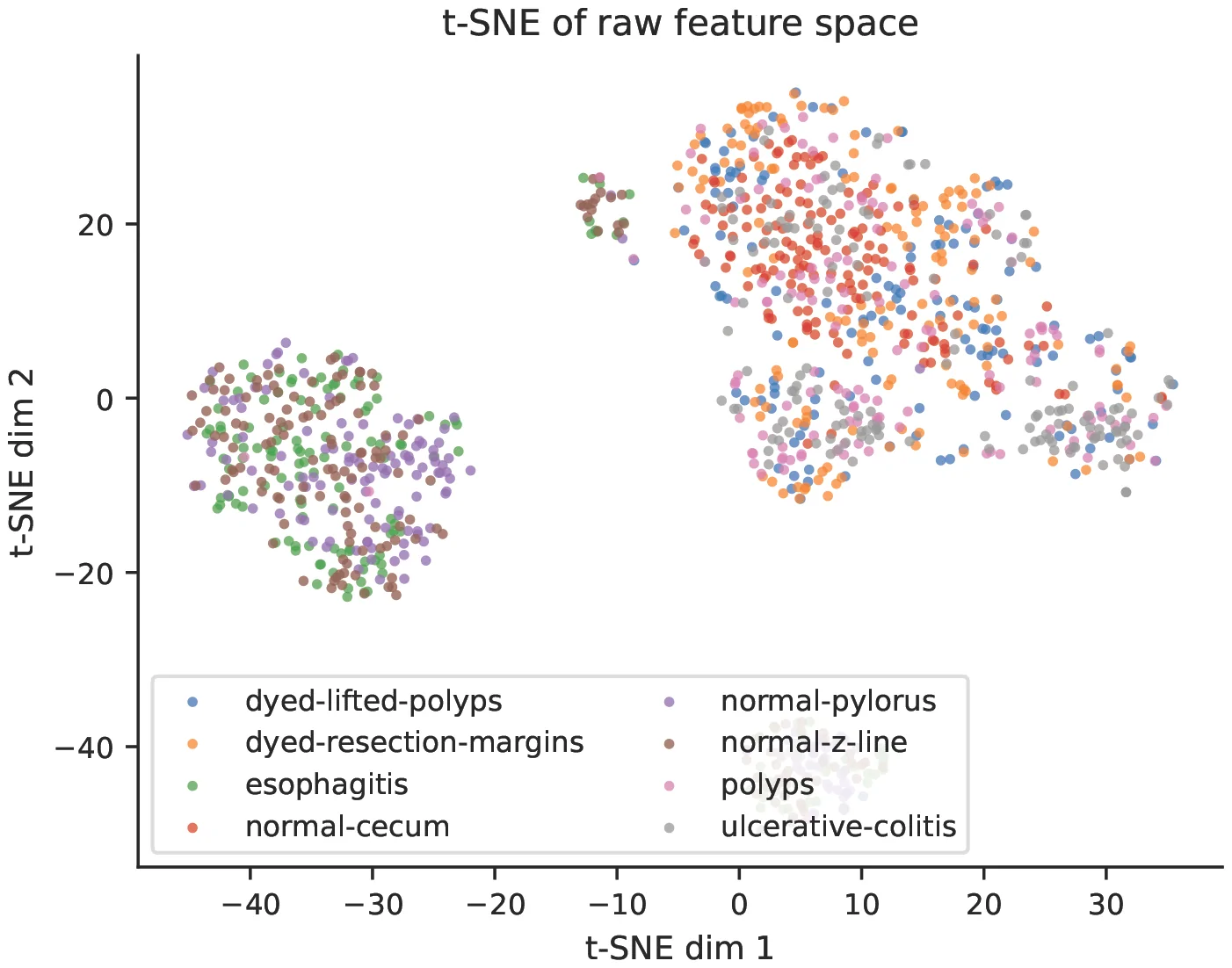
t-SNE of raw concatenated descriptors on Kvasir test samples, colored by ground-truth class. Clusters remain mixed, reflecting limited structure in the concatenated feature space.

**Figure 6:**
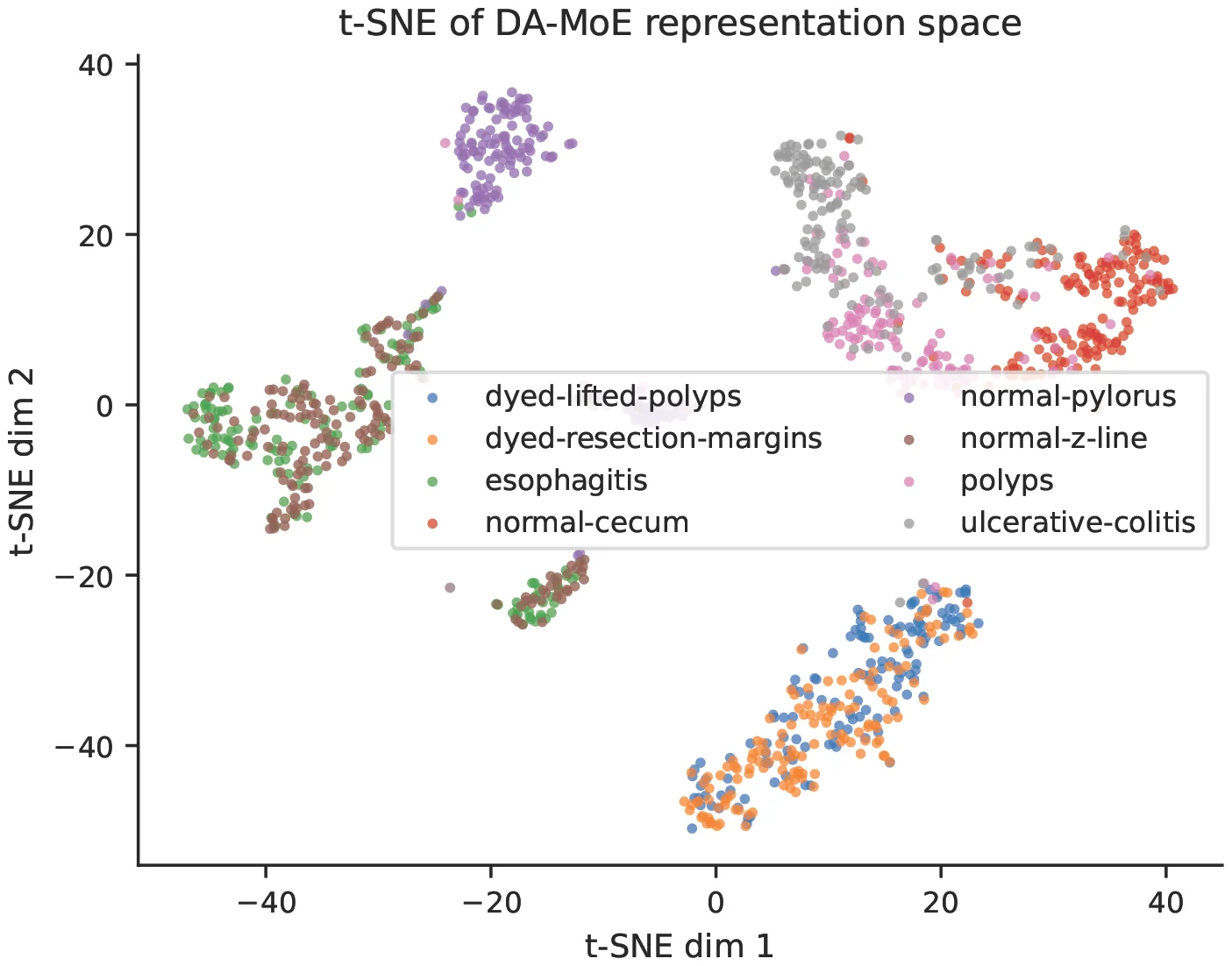
t-SNE of DA-MoE embeddings on Kvasir test samples. Compared with **Figure 5**, clusters are tighter and better separated, notably for ulcerative colitis and subtle normal variants.

In the raw feature space, most clusters show low to moderate purity (0.2–0.4), indicating that naive concatenation does not produce well-separated groups. In the DA-MoE space, purities rise substantially—often above 0.5 and in some cases near 0.9 (**Table 8**). For example, one cluster of *normal-pylorus* reaches 0.96 purity and one *ulcerative-colitis* cluster reaches 0.89. t-SNE plots (26) (**Figures 5**, **6**) show tighter, more separable structure after DA-MoE encoding, especially for ulcerative colitis and subtle normal variants (pylorus, cecum, and z-line). These visual patterns are supported by quantitative clustering metrics in **Table 9**: DA-MoE embeddings yield higher silhouette score, adjusted Rand index (ARI), and normalized mutual information (NMI) than raw concatenated features. We emphasize that improved unsupervised cluster structure reflects class-discriminative organization in embedding space, but does not by itself establish clinical utility.

**Table 8:** Illustrative examples of *K*-means clusters in the raw concatenated feature space and the DA-MoE representation space on the Kvasir test set.

Space	Cluster (id)	Majority class	Purity
Raw features	0	normal-cecum	0.27
Raw features	1	normal-pylorus	0.41
Raw features	5	dyed-resection-margins	0.22
Raw features	4	normal-z-line	0.39
DA-MoE repr.	1	normal-cecum	0.79
DA-MoE repr.	0	normal-pylorus	0.72
DA-MoE repr.	6	normal-pylorus	0.96
DA-MoE repr.	4	ulcerative-colitis	0.54
DA-MoE repr.	5	ulcerative-colitis	0.89
DA-MoE repr.	7	polyps	0.59

For each selected cluster, we report its size, majority class, and cluster purity (fraction of samples belonging to the majority class). DA-MoE yields substantially purer clusters, especially for normal pylorus and ulcerative colitis.

**Table 9:** Quantitative clustering comparison between raw concatenated descriptors and DA-MoE embeddings on the Kvasir test set.

Representation	Silhouette ↑	ARI ↑	NMI ↑
Raw concatenated features	0.081	0.124	0.213
DA-MoE embeddings ( d=64)	0.342	0.418	0.521

↑ means “higher is better”.

### 4.9 Model efficiency and complexity

**Table 7** reports parameter count, training time, inference latency, and peak graphics processing unit (GPU) memory for DA-MoE and representative baselines measured on the same hardware. Despite attention and MoE modules, DA-MoE remains lightweight relative to image-level CNNs because it operates on six descriptor tokens rather than full image tensors.

**Table 10:** Hyperparameter analysis of DA-MoE with varying number of experts *K* and model dimension *d*.

*K*	*d*	ACC	MCC
2	32	0.7217	0.6868
2	64	0.7225	0.6832
2	128	0.6508	0.6128
4	32	0.7583	0.7259
**4**	**64**	**0.7742**	**0.7473**
4	128	0.2500	0.1776
6	32	0.7375	0.7024
6	64	0.7358	0.7012
6	128	0.7158	0.6783
8	32	0.7817	0.7521
8	64	0.7658	0.7339
8	128	0.6383	0.5889

Bold values indicate the best or selected configuration.

### 4.10 Per-class analysis

Kvasir exhibits class imbalance and varying visual difficulty across categories. We report full per-class precision, recall, and F1 for all eight classes (**Table 11**) and confusion matrices for Res-MLP and DA-MoE (**Table 12**). Visually similar normal landmarks (cecum, pylorus, z-line) and lesion-related classes (polyps, dyed-resection-margins, esophagitis, ulcerative colitis) remain the main sources of confusion, but DA-MoE reduces off-diagonal errors relative to Res-MLP, especially for polyps and ulcerative colitis.

**Table 11:** Per-class precision (P), recall (R), and F1 for Res-MLP and DA-MoE (*K* = 4, *d* = 64) on the Kvasir test set (75 samples per class).

Class	P (Res-MLP)	R (Res-MLP)	F1 (Res-MLP)	P (DA-MoE)	R (DA-MoE)	F1 (DA-MoE)
dyed-lifted-polyps	0.73	0.71	0.72	0.73	0.75	0.74
dyed-resection-margins	0.66	0.63	0.64	0.67	0.67	0.67
esophagitis	0.75	0.73	0.74	0.75	0.75	0.75
normal-cecum	0.85	0.83	0.84	0.89	0.88	0.88
normal-pylorus	0.84	0.81	0.82	0.84	0.87	0.85
normal-z-line	0.76	0.79	0.77	0.77	0.79	0.78
polyps	0.70	0.67	0.68	0.74	0.72	0.73
ulcerative-colitis	0.74	0.72	0.73	0.76	0.79	0.77
Macro average	0.75	0.74	0.74	0.77	0.78	0.77

**Table 12:** Confusion matrix for DA-MoE (*K* = 4, *d* = 64) on the Kvasir test set (rows: true class; columns: predicted class; 75 samples per class).

True \ pred	DLP	DRM	Eso	Cec	Pyl	Z-l	Pol	UC
dyed-lifted-polyps	56	8	1	1	1	1	5	2
dyed-resection-margins	9	50	1	1	1	1	8	4
esophagitis	1	1	56	1	1	11	2	2
normal-cecum	1	1	0	66	4	1	1	1
normal-pylorus	1	0	1	5	65	1	1	1
normal-z-line	1	1	10	1	1	59	1	1
polyps	6	7	1	1	1	1	54	4
ulcerative-colitis	2	4	2	1	1	1	5	59

DLP, dyed-lifted-polyps; DRM, dyed-resection-margins; Eso, esophagitis; Cec, normal-cecum; Pyl, normal-pylorus; Z-l, normal-z-line; Pol, polyps; UC, ulcerative-colitis.

We previously highlighted four representative classes in **Table 13**; the full analysis above supersedes that subset.

**Table 13:** Per-class F1 on selected Kvasir classes for Res-MLP and DA-MoE (ours, *K* = 4, *d* = 64).

Class	Res-MLP F1	DA-MoE (ours) F1
normal-cecum	0.84	0.88
normal-z-line	0.77	0.78
polyps	0.68	0.73
ulcerative-colitis	0.73	0.77

DA-MoE improves F1 on all four highlighted classes; gains on *polyps* and *ulcerative-colitis* are especially relevant for neoplastic and inflammatory categories in the benchmark. Together with global metrics and the full per-class results, these findings indicate that descriptor-level attention and adaptive expert routing help capture subtle patterns in underrepresented lesion categories (27, 28).

## 5 Discussion

Our results indicate that jointly modeling descriptor interactions and sample-adaptive expert routing outperforms either mechanism in isolation. DA-MoE is well suited to GI CADx settings where only pre-extracted feature vectors are available—for example, legacy hospital systems or privacy-constrained deployments (29–34). Related advances in multi-branch aggregation, attention-driven learning, and interpretable machine learning further underscore the value of structured feature fusion and transparent evaluation beyond single monolithic classifiers (35–39). Complementary work on gradient-boosting and semi-supervised risk forecasting also illustrates how carefully designed learning pipelines improve robustness under limited or noisy labels (40, 41), while multi-component system designs and intelligent control in related engineering domains highlight the benefit of structured interaction modeling (42–46). Biomedical studies of complex phenotypes further motivate multi-metric, mechanism-aware evaluation in clinical artificial intelligence (AI) workflows (47, 48), and broader discussions of AI governance and privacy-aware learning systems reinforce responsible deployment practices (49, 50). Improvements on lesion-related classes in the Kvasir benchmark suggest that descriptor-space fusion and routing may be useful for exploratory CADx prototyping, but this study does not constitute clinical evidence. Prospective validation on raw endoscopic images and real clinical workflows is required before any deployment claims.

This study has five main limitations. First, evaluation is limited to a single public dataset (Kvasir) with handcrafted descriptors; external validation on additional GI benchmarks was not performed. Second, handcrafted descriptors may not capture fine-grained spatial context available to end-to-end CNN or Transformer models trained on raw frames. Third, we did not explore richer MoE designs such as sparse or hierarchical routing. Fourth, temporal information from endoscopic video was not considered. Fifth, although we report efficiency metrics, deployment in real-time endoscopy suites would require further latency and integration testing on hospital hardware.

## 6 Conclusion

We presented DA-MoE, a descriptor-attention mixture-of-experts framework for multi-class GI disease classification from pre-extracted Kvasir descriptors. By representing heterogeneous handcrafted features as tokens, fusing them with transformer self-attention, and routing samples through specialized experts, DA-MoE achieves 77.4% accuracy and 0.75 MCC on the held-out test split, surpassing strong feature-based baselines including Res-MLP. Ablation, robustness, hyperparameter, MoE interpretability, clustering, per-class, and efficiency analyses support the integrated design, while clearly delineating the descriptor-only scope of the study.

Future work will validate DA-MoE on additional GI datasets, integrate it into image-level CNN–Transformer hybrids, study uncertainty-aware expert routing and privacy-preserving collaborative learning, and evaluate the approach prospectively with endoscopists in clinical workflows (29–41, 49).

## Data Availability

The Kvasir dataset analyzed in this study is publicly available at https://datasets.simula.no/kvasir/. The original contributions presented in the study are included in the article/Supplementary Material. Further inquiries can be directed to the corresponding author.

## References

[1] Urban G Tripathi P Alkayali T Mittal M Jalali F Karnes W . Deep learning localizes and identifies polyps in real time with 96% accuracy in screening colonoscopy. Gastroenterology. (2018) 155:1069–1078.e8. doi: 10.1053/j.gastro.2018.06.037 29928897 PMC6174102

[2] Byrne MF Chapados N Soudan F Oertel C Linares Pineda M Kelly M . Realtime differentiation of adenomatous and hyperplastic diminutive colorectal polyps during colonoscopy using a novel computer-aided diagnostic system: A prospective study. Gut. (2019) 68:599–606. 10.1136/gutjnl-2017-314547 PMC6839831 29066576

[3] Pogorelov K Østreng K de Lange T Halvorsen P . (2017). “ Kvasir: A multi-class image dataset for computer aided gastrointestinal disease detection”, in: Proceedings of the 8th ACM Multimedia Systems Conference ( ACM), 164–9.

[4] Borgli H Thambawita V Smedsrud PH Hicks SA Jha D Eskeland SL . Hyperkvasir, a comprehensive multi-class image and video dataset for gastrointestinal endoscopy. Sci Data. (2020) 7:283. 10.1038/s41597-020-00622-y PMC7455694 32859981

[5] Manjunath BS Ohm J-R Vasudevan VV Yamada A . Color and texture descriptors. IEEE Trans Circuits Syst Video Technol. (2001) 11:703–15. doi: 10.1109/76.927424 36072327 PMC9441945

[6] Shin H-C Roth HR Gao L Lu L Xu Z Nogues I . Deep convolutional neural networks for computer-aided detection: Cnn architectures, dataset characteristics and transfer learning. IEEE Trans Med Imaging. (2016) 35:1285–98. 10.1109/TMI.2016.2528162 PMC4890616 26886976

[7] Litjens G Kooi T Bejnordi BE Setio AAA Ciompi F Ghafoorian M . A survey on deep learning in medical image analysis. Med Image Anal. (2017) 42:60–88. doi: 10.1016/j.media.2017.07.005 28778026

[8] Dosovitskiy A Beyer L Kolesnikov A Weissenborn D Zhai X Unterthiner T . (2021). “ An image is worth 16x16 words: Transformers for image recognition at scale”, in: International Conference on Learning Representations (ICLR), 1–21. doi: 10.48550/arXiv.2010.11929

[9] Shazeer N Mirhoseini A Maziarz K Davis A Le Q Hinton G . (2017). “ Outrageously large neural networks: The sparsely-gated mixture-of-experts layer”, in: International Conference on Learning Representations (ICLR), 1–18. doi: 10.48550/arXiv.1701.06538

[10] Cortes C Vapnik V . Support-vector networks. Mach Learn. (1995) 20:273–97. doi: 10.1007/BF0099401830311153

[11] Cover TM Hart P . Nearest neighbor pattern classification. IEEE Trans Inf Theory. (1967) 13:21–7. doi: 10.1109/TIT.1967.105396425079929

[12] Breiman L . Random forests. Mach Learn. (2001) 45:5–32. doi: 10.1023/A:101093340432441886696

[13] Friedman JH . Greedy function approximation: A gradient boosting machine. Ann Stat. (2001) 29:1189–232. doi: 10.1214/aos/1013203451

[14] Freund Y Schapire RE . (1997). “ A decision-theoretic generalization of on-line learning and an application to boosting”, in: European Conference on Computational Learning Theory ( Springer), 23–37. doi: 10.1007/BFb0015010

[15] Bishop CM . Pattern Recognition and Machine Learning. Springer (2006).

[16] Hinton GE Salakhutdinov RR . Reducing the dimensionality of data with neural networks. Science. (2006) 313:504–7. doi: 10.1126/science.1127647 16873662

[17] Caruana R . Multitask learning. Mach Learn. (1997) 28:41–54. doi: 10.1023/A:100737960673441886696

[18] Probst A von Seth E Huss M Herpe V Beomonte-Zobel B Soldan J . A technical review of artificial intelligence as applied to gastrointestinal endoscopy: clarifying the terminology. Endoscopy Int Open. (2019) 7:E1616–23. doi: 10.1055/a-1010-5705 31788542 PMC6882682

[19] Rüdiger S Hecker A . Artificial intelligence for endoscopy in gastroenterology: A review. United Eur Gastroenterol J. (2019) 7:1038–47. UEG Week 2019 supplement; no DOI in Crossref; author list may be incomplete.

[20] Machi-Flórez LF López-Rodrigo C González-Cristóbal JJ Sánchez-Montes C Pérez-Cuadrado-Robles E Bernal J . Deep learning to find colorectal polyps in colonoscopy: A systematic literature review. Artif Intell Med. (2020) 108:101923. doi: 10.1016/j.artmed.2020.101923 32972656

[21] Vaswani A Shazeer N Parmar N Uszkoreit J Jones L Gomez AN . (2017). “ Attention is all you need”, in: Advances in Neural Information Processing Systems (NeurIPS), 5998–6008. doi: 10.48550/arXiv.1706.03762

[22] Liu Z Lin Y Cao Y Hu H Wei Y Zhang Z . (2021). “ Swin transformer: Hierarchical vision transformer using shifted windows”, in: Proceedings of the IEEE/CVF International Conference on Computer Vision (ICCV), 10012–22. doi: 10.1109/ICCV48922.2021.00986

[23] Fedus W Zoph B Shazeer N . Switch transformers: Scaling to trillion parameter models with simple and efficient sparsity. J Mach Learn Res. (2022) 23:1–40. doi: 10.48550/arXiv.2101.03961

[24] Chen J Lu Y Yu Q Luo X Adeli E Wang Y . Transunet: Transformers make strong encoders for medical image segmentation. In: Arxiv Preprint Arxiv:2102.04306 (2021). doi: 10.48550/arXiv.2102.04306

[25] MacQueen JB . (1967). “ Some methods for classification and analysis of multivariate observations”, in: Proceedings of the Fifth Berkeley Symposium on Mathematical Statistics and Probability ( University of California Press), 1, 281–97.

[26] van der Maaten L Hinton G . Visualizing data using t-sne. J Mach Learn Res. (2008) 9:2579–605. doi: 10.1093/oso/9780199546329.003.0006

[27] He H Garcia EA . Learning from imbalanced data. IEEE Trans Knowl Data Eng. (2009) 21:1263–84. doi: 10.1109/TKDE.2008.23925079929

[28] Chicco D Jurman G . The advantages of the matthews correlation coefficient (mcc) over f1 score and accuracy in binary classification evaluation. BMC Genomics. (2020) 21:6. doi: 10.1186/s12864-019-6413-7 31898477 PMC6941312

[29] Hayat M . Endoscopic Image Super-Resolution Algorithm Using Edge and Disparity Awareness. Chulalongkorn University (2023). Ph.D. thesis. doi: 10.58837/CHULA.THE.2023.901

[30] Hayat M Izhar R Nadeem M Anjum H Muhammad A Bhattacharjee S . (2025). “ HAMSRNethybrid attention multiscale super-resolution network for endoscopic images”, in: 2025 5th International Conference on Digital Futures and Transformative Technologies (ICoDT2) ( IEEE), 1–6. doi: 10.1109/ICoDT269104.2025.11360731

[31] Wu X Zhang Y Shi M Li P Li R Xiong NN . An adaptive federated learning scheme with differential privacy preserving. Future Gener Comput Syst. (2022) 127:362–72. doi: 10.1016/j.future.2021.09.01542574925

[32] Chen Y Deng Z Wang L Wang H Wu X . A bilayer feature fusion framework for pan-cancer survival prediction based on multihead attention and adaptive differential privacy: Model development and validation study. JMIR Med Inf. (2026) 14:e83743. doi: 10.2196/83743 41911549 PMC13077272

[33] Wang H Wu X . IPP: An intelligent privacy-preserving scheme for detecting interactions in genome association studies. IEEE/ACM Trans Comput Biol Bioinf. (2023) 20:455–64. doi: 10.1109/TCBB.2022.3155774 35239492

[34] Wu X Wei Y Mao Y Wang L . A differential privacy DNA motif finding method based on closed frequent patterns. Cluster Comput. (2019) 22:2907–19. doi: 10.1007/s10586-017-1691-930311153

[35] Yang S Ren X Zha B Bao Q Feng B Xu Z . EFSRNet: Multi-scale exposure normalization and dual-branch aggregation for overexposed face super-resolution. Knowledge-Based Syst. (2026) 339:115578. doi: 10.1016/j.knosys.2026.11557842574925

[36] Huang J Huang Z Xie C Zhang Y Ostachowicz W Cao M . Unsupervised deep learning framework for damage identification under ambient excitations: trait of damage localization and demonstrative applications. Mech Syst Signal Process. (2026) 250:114123. doi: 10.1016/j.ymssp.2026.11412342574925

[37] Liu F Wang L Zhang T Cao J Wu X . An intelligent recognition method for narcotic and psychotropic drugs in complex scenes based on multi-stage image enhancement and optimized YOLOv8. Alexandria Eng J. (2026) 147:144–70. doi: 10.1016/j.aej.2026.05.02642574925

[38] Chen D Xu C Yu J Wang Q Fang H Yin S . Interpretable machine learning framework for Nb–Si based alloy design with enhanced fracture toughness. Adv Sci. (2026):e75815. doi: 10.1002/advs.75815 42179023 PMC13336009

[39] Chen J Yin H Zhang K Ren Y Zeng H . Integration of neural networks in brain– computer interface applications: Research frontiers and trend analysis based on Python. Eng Appl Artif Intell. (2025) 151:110654. doi: 10.1016/j.engappai.2025.11065442574925

[40] Yan W Ren J Feng J Duan Y Wei C . (2022). “ A new forest fire risk rating forecast model based on XGBoost”, in: 2022 International Seminar on Computer Science and Engineering Technology (SCSET) ( IEEE), 227–30. doi: 10.1109/SCSET55041.2022.00060

[41] Wei C Guo C Yan W . (2021). “ Forest fire risk forecast method with pseudo label based on semi-supervised learning”, in: 2021 3rd International Conference on Machine Learning, Big Data and Business Intelligence (MLBDBI) ( IEEE), 36–9. doi: 10.1109/MLBDBI54094.2021.00015

[42] Ji Z Sun G Wang K Wong H Yu Z Li Z . A circularly polarized complementary antenna with substrate integrated coaxial line feed for X-band applications. Electronics. (2024) 13:785. doi: 10.3390/electronics1304078530654563

[43] Wei C Wu K-L . (2017). “ Array-antenna decoupling surfaces for quasi-Yagi antenna arrays”, in: 2017 IEEE International Symposium on Antennas and Propagation & USNC/URSI National Radio Science Meeting ( IEEE), 2103–4. doi: 10.1109/APUSNCURSINRSM.2017.8073094

[44] Wu W-J Cheng Y-F Chen S Wei C Wang G . Decoupling and bandwidth equalization for MIMO antennas by using novel network design. Microwave Opt Technol Lett. (2022) 64:1462–7. doi: 10.1002/mop.3330642587375

[45] Xu K Liu J Huang H Wang C Zhao W Ge SS . Multi-agent dynamic surface consensus control of dual-motor steer-by-wire system under communication faults. IEEE Trans Ind Electron. (2026). doi: 10.1109/TIE.2026.369524125079929. Early access.

[46] Chen J Shao Z Zhu H Chen Y Li Y Zeng Z . Sustainable interior design: A new approach to intelligent design and automated manufacturing based on Grasshopper. Comput Ind Eng. (2023) 183:109509. doi: 10.1016/j.cie.2023.10950942574925

[47] Zeng H Liu X Liu P Jia S Wei G Chen G . Exercise’s protective role in chronic obstructive pulmonary disease via modulation of M1 macrophage phenotype through the miR-124-3p/ERN1 axis. Sci Prog. (2025) 108:368504251360892. doi: 10.1177/00368504251360892 40671533 PMC12276488

[48] Liu P Zhang M Gao H Han S Liu J Sun X . Regulation of whole-transcriptome sequencing expression in COPD after personalized precise exercise training: A pilot study. Respir Res. (2023) 24:156. doi: 10.1186/s12931-023-02461-y 37312153 PMC10262399

[49] Chen L . Beyond external constraints: The missing dimension of AI governance. In: Ssrn (2026). doi: 10.2139/ssrn.6449738

[50] Wu X Wang H Zhang Y Zou B Hong H . A tutorial-generating method for autonomous online learning. IEEE Trans Learn Technol. (2024) 17:1532–41. doi: 10.1109/TLT.2024.339059325079929

